# Indigenous Remedies of the Southern States

**Published:** 1868

**Authors:** Joseph Jones

**Affiliations:** Professor of Physology and Pathology in the Medical Department of the University of Nashville, Tenn.


### ARTICLE IV.

### *Indigenous Remedies of the Southern States which may be Employed as Substitutes for Sulphate of Quinine in the Treatment of Malarial Fever.* By JOSEPH JONES, M.D., Professor of Physology and Pathology in the Medical Department of the University of Nashville, Tenn.

### NO. 2.—DOGWOOD (CORNUS FLORIDA.)

*Botanical Description—Geographical Distribution—Chemical Composition—Examination of by Dr. Walker, of Virginia,* 1803—*Dr. Walker's Receipt for making Ink from the Bark—Examination of by Mr. Carpenter, of Philadelphia—Cornine—Observations of Drs. Staples, S. Jackson, James Cockburn, D. C. O'Keefe—Medical Properties and Uses—Testimony of Dr. Walker, of Virginia, to the Medical Properties of Dogwood, of Dr. Gray, of Bristol, of Dr. Jacob Bigelow, S. G. Morton, R. Coates, D. C. O'Keefe, and others—Method of Preparing the Extract Dose—Experience of Author with in Southern Army During War,* 1861–65; *and, as a Substitute for Quinine, Possesses Prophylactic Powers against Malarial Fever—Efforts of Surgeon-General Moore and of Medical Purveyors to supply Innigenous Remedies—Testimony of Assistant Surgeon Warren and of Surgeon F. P. Porcher to the Medical Properties of Dogwood.*

*Botanical Character.*—Arborescent; leaves ovate, acuminate; involucrum large; obcordate; drupes ovate. A tree fifteen to twenty-five feet high, the trunk eight to ten inches diameter, with expanding branches, the smaller crowded at the extremities of the older. Wood fine grained, hard, durable. Leaves opposite, deciduous, ovate, lanceolated, acuminate, entire, ribbed; the younger ones very pubescent, almost villous on the under surface. Flowers in terminal heads. Involucrum four leaved; leaves large, obcordate, nerved, white; the serius callous, sessile at the base of each head, and enclosing it before the time of flowering. Calyx one leaved, small, tubular, border four cleft; segments erect, obtuse, shorter than the tube. Petals four, linear, lanceolate, inserted into the summit of the germ, yellowish. Filaments four, as long as the corolla, alternating with the petals. Anthers incumbered, two lobed. Germ inferior, slightly angled. Style shorter than the stamers, surrounded at base by a glandular ring, around which the petals and filaments are inserted. Stigma capitate. Drupe red. Flowers March to April.—ELLIOTT. *Sketch of Botany of South Carolina and Georgia. Vol.* 1, *pp.* 207–208.

*Geographical Distribution.*—The Cornus Florida is first seen in Massachusetts, between the 42d deg. and 43d deg. of latitude, and extends uninterruptedly throughout the Eastern, Southern and Western States to the banks of the Mississippi. Although abounding especially in the Middle States, it is, nevertheless, one of the most common trees over this vast extent of country. In New Jersey, Pennsylvania, Maryland and Virginia it abounds upon moist, gravelly and uneven soil. In North Carolina, South Carolina, Georgia, Florida and Alabama it is generally found most abundant and most luxuriant on the borders of swamps and low grounds, and scarcely ever in the pine barrens, where the soil is too dry and sandy to sustain any trees but the long leaf Pine (Pinus Australis), the Barren Scrub Oak (Quercus catesbæi), Upland Willow Oak, (Quercus cinerea), Black Jack Oak (Quercus ferruginea), and Running Oak (Quercus pumila). In the most fertile districts of West Tennessee and Kentucky it is said not to appear in the forests except where the soil is gravelly and of middling quality.

*Chemical Composition.*—The bark of the root, stem and branches of the Cornus Florida is a powerful bitter, possessing a bitter astringent and slightly aromatic taste. The chemical composition of this bark appears to have been first investigated by Dr. Walker, of Virginia, who published his observations in Philadelphia.* He found that water distilled from the bark in powder had a transparent, whitish appearance, with a slight aromatic odor, and no perceptible taste. When the heat was increased, the fluid had a lemon color, with an unpleasant smell and an acerb taste, effects which were probably produced by the volatilization and partial decomposition of portions of the bark in consequence of the continuance of the heat until the moisture was evaporated nearly to dryness. Dr. Walker also endeavored to ascertain the effects of different menstrua upon the extract furnished by evaporating a decoction of the root of Cornus Florida. Strong alcohol dissolved from the extract three-fourths of the entire quantity; the part which remained undissolved was destitute of taste, and underwent no change of color on adding the test of iron. The alcohol which contained the dissolved portion of the extract possessed an intensely bitter taste, with astringency; presented a clear, red color, and turned to a deep black on the addition of a salt of iron. When the alcohol extract was macerated in repeated portions of sulphuric ether, with a view to ascertain the quantity of resin, the ether acquired a dark color and a bitter taste, and dissolved three-quarters of the extract. When tested with iron it was found that the remaining quarter only was changed to a black color.† Upon the examination

* Experimental inquiry into the similarity in virtue between the Cornus Florida and Sericea, and the Cinchona Officinalis of Linnæns, &c., &c. By Dr. John M. Walker. Philadelphia. 1803.

† Dr. Walker gives a receipt for making an excellent ink, in which the bark of the Cornus Florida is substituted for gall nuts: Put half an ounce of dogwood bark, two scruples of sulphate iron and two scruples of gum arabic in sixteen ounces of rain water. During the infusion shake it repeatedly.

Dr. Walker announced that the dogwood contained gun, resin, tannin, and gallic acid. Dr. Walker thus sums up the results of his experiments: "A summary recapitulation of these experiments shows that the Cornus Florida and sericea and the Peruvian bark possess the same ingredients—that is, gum, mucilage and extract, which last contains the tannin and gallic acid, though in different proportions. The Florida possesses most of the gum, mucilage and extract; the sericea next, which appears to be an intermediate between the Florida and Peruvian bark; while the latter possesses most of the resin."

The virtues appear equally similar in their residue. The extract and resin possess all their active virtues. The extract appears to possess all their tonic power. The resin, when perfectly separated from the extract, appears to be purely stimulant, and probably the tonic power of the extract is increased when combined with a portion of the resin, as in the spirituous tincture.

Mr. G. W. Carpenter, of Philadelphia,* subsequently announced the discovery of a peculiar bitter principle, for which he proposed the name cornine, and which he asserted to be the active alkaloid principle of the Cornus Florida, and to be fully equal, if not superior, to quinine in its tonic and febrifuge properties. In consequence, however, of yielding this salt in so very minute comparative proportion to what the quinine is yielded by the cinchona, it is even more expensive than the latter. It is greatly to be regretted that Mr. Carpenter did not publish the method by which he extracted this alkaloid principle. Some have even gone so far as to affirm that he did not discover any alkaloid principle at all, because subsequent investigations have failed to detect cornine. We conceive this criticism to be entirely too severe, for three reasons:

1. No absolutely accurate and complete examination of the bark of the Cornus Florida has been made.

2. As Mr. Carpenter did not state his method of obtaining the active principle, it might be supposed that the reagents used have exerted some influence in the transformation as well as the separation of the alkaloid principle.

3. Mr. Carpenter affirms that he submitted the alkaloid cornine to the examination of several physicians.

---

*Essays on some of the most important articles of the Materia Medica, &c. By G. W. Carpenter. Philadelphia. 1834. Page 202.

This subject is of so much interest and importance that we quote the entire passage from the work of Mr. Carpenter:

"It gives me much pleasure to announce the discovery which I made of an alkaloid base in the Cornus Florida, which I have denominated Cornine, and which, with acids, forms neutral salts, the sulphate of which has proved a highly valuable tonic and febrifuge. This article has been very carefully and accurately described by Dr. Samuel G. Morton, of this city, in the *Philadelphia Journal of the Medical and Physical Sciences*, and from the most respectable sources in the medical profession from various parts of the United States where this article has been sent, the most corroborating evidences have been received of the unequivocal success of the Cornine in the treatment of intermittent and remittent fevers, in the same doses as the quinine; and the only circumstance which precludes its competion with that substance is the minute comparative proportion of Cornine yielded by the Cornus Florida. If, however, at any time we should fail in our supplies of Cinchona, which is not impossible, or even improbable, we shall then be able to supply its place by this principle of the Cornus Florida." —*Essays on the most important Articles of the Materia Medica. Page* 203.

Dr. S. G. Morton,* of Philadelphia, described cornine as a grayish-white powder, extremely bitter, and deliquescent when exposed to the air, and affirmed that he had exhibited it in cases of intermittent fever with much success. Dr. Morton considered it to be in no respect inferior to quinine.

Dr. R. Coates, and several other practitioners, exhibited this salt in the same cases in which sulphate of quinine is employed, and with decided success.

Cornia, according to Mr. Carpenter, does not crystalize, but forms, on evaporation, a viscid mass. It is a pale straw color, attracts the moisture of the atmosphere, and dissolves in alcohol and in sulphuric, acetic and muriatic acids, with which it forms crystallizable neutral salts. The sulphate crystallizes in acicular or needle-like crystals, deliquescent, and consequently soluble in water, of a grayish-white color, and its taste is intensely bitter. According to the testimony of Joseph Tongo,† M.D., and E. Durand, of Philadelphia,

* Philadelphia Journal of the Medical and Physical Sciences.

†A Manual of Materia Medica and Pharmacy, comprising a concise description of the articles used in Medicine. By H. M. Edwards, M.D., and P. Vavasseur, M.D. Translated from the French by Joseph Tongo, M.D., &c., and E. Durand, &c. Philadelphia, 1829.

Dr. Staples obtained it by digesting the bark of the root of the Cornus Florida in alcohol of 30 deg. of Paumé's areometer. After several days had elapsed, the latter was filtered and concentrated by distillation in a water bath. On cooling a granular extract was obtained, of a light pink color, of a very bitter and astringent taste; when treated with diluted sulphuric acid, afforded a very small quantity of crystals of sulphate of cornia, without having been exhausted of all its bitterness and astringency.

Mr. Ellis states that Dr. S. Jackson, lately of Northumberland, Pennsylvania, informed him that he had subjected the bark to Henry's process for obtaining quinine from cinchona, and that without carrying the process so far as to obtain a crystalline salt, he used the concentrated alcoholic solution with most decisive results, and was satisfied that it contained a principle analogous to quinia.

Mr. James Cockburn examined the Cornus Florida in 1835, with the following results:

The decoction, which was of a light red color, and slight mucilaginous appearance, formed a precipitate with a solution of subacetate of lead, which consisted of gum, coloring matter, and other foreign substances. A precipitate was also formed with pure alcohol. Upon the addition of water to the tincture, concentrated by evaporation, it threw down a curdy precipitate, which, upon examination, was found to be resin.

The decoction and tincture redden litmus paper, and cause a yellowish precipitate in a solution of gelatine, and one of a dark olive green in a solution of sulphate of iron. They also afford precipitates with sulphuric and muriatic acids, lime water, alumina, the carbonates of ammonia and potassa, tartrate of antimony and potassa. The color becomes lighter on the addition of nitric acid, milky by the corrosive chloride of mercury, and has its color deepened by ammonia.

A portion of the bark was digested in sulphuric ether for a few days and filtered. The ethereal tincture was of a lemon color, and reddened litmus paper, and on evaporation deposited on the sides of the vessel a fatty matter, insoluble in water, but soluble in alcohol, leaving a greasy stain on paper. Besides this there was a compound of oil and resin combined with coloring matter, and a substance of a light brown color, very bitter taste, friable and very regular appearance, supposed to be a compound of a peculiar bitter principle, mixed with tannin and other matters. This was dissolved in alcohol, and formed a beautiful red colored tincture, which reddened litmus paper. A substance resembling the ethereal residue remained, interspersed with small, shining acicular crystals, of a bitter taste, which property I am disposed to believe they owed to the bitter extract with which they were associated. The bark used in the last experiment was submitted to the action of boiling ether, which, on cooling, deposited a substance of the consistence of wax, which it resembled in all its properties.

Two ounces of the bark, coarsely powdered, were then introduced into ℥ viii. of alcohol, and exposed to a temperature of from 105 deg. to 120 deg. F. The alcohol was then decanted, and a fresh portion added and treated as before. The liquors were then united, and a solution of subacetate of lead added to separate the coloring matter. After the insoluble portion subsided the clear liquor was separated, a little sulphuric acid was then added to the solution to separate any excess of subacetate of lead. This was filtered and the alcohol distilled off. There remained in the retort only an oily-like substance, together with a principle of a dirty white color, curdled appearance, resembling the residue of the ethereal tincture. Ammonia was then added to the liquor to precipitate any principle remaining in solution. The residue was then treated with a little sulphuric acid, water and animal charcoal (previously treated with muriatic acid), which, upon evaporation, deposited an abundant crystalline mass, of a flaky appearance, resembling at first sulphate of quinine, but on cooling assumed a feathery appearance, with a sharp, saline taste, soluble in hot and cold water, insoluble in alcohol and ether, soluble in nitric acid, and resembled sulphate of ammonia in all its properties.

One pound of coarsely powdered bark was boiled for half an hour in one gallon of water, acidulated with ʒ iss. sulphurid acid. The tincture was poured off and treated with animal charcoal, and when evaporated left a brown extract of a resinous, waxy appearance, and very bitter taste, which appeared to have very much the the flavor of Peruvian bark. This was again treated with animal charcoal, and left, on evaporation, a crystalline mass in an impure form, which was slightly soluble in alcohol, almost insoluble in ether, but very soluble in nitric acid. The alcoholic solution was evaporated, and left crystals of a very fine, long, flexible and silky appearance, which crystals decomposed when thrown upon red coals, and did not form a precipate with oxalate of ammonia, but were without taste. The bitterness was entirely owing to the bitter extract, which was slightly soluble in water, soluble in alcohol, but nearly insoluble in ether. This I propose to call bitter extractive; and in this, I am inclined to believe, the active principle resides.

A concentrated tincture yielded by evaporation a dark brown extract, slightly soluble in water, soluble in alcohol and ether, bitter aromatic taste, possessing the properties of resin. Both this and the watery extract possess the sensible properties of the bark in a concentrated form.

There is a red coloring principle in this bark, taken up very feebly by alcohol and ether, but less so by water, and has its color rendered deeper by an alkali.

One thousand grains of the bark yielded, by incineration, a product weighing sixty-five grains. This residue was submitted to the action of boiling water, and concentrated by evaporation; it then had an alkaline taste, effervescent strongly with acids, and restored the blue color to litmus, previously reddened by an acid. It was then neutralized with nitric acid, and upon evaporation yielded crystals of nitrate of potassa.

The insoluble residue of the preceding experiment was dissolved by nitric acid (with the exception of a minute portion of carbonaceous matter), with violet effervescence. The colorless solution thus obtained threw down a white precipitate, on the addition of oxalate of ammonia, and a deep blue one with ferrocyanate of potassa. It produced also a dark green or black with tincture of galls. Carbonate of soda, when added to the solution, caused a white flocculent precipitate. On adding a solution of phosphate of soda, no change was immediately produced, which led to the belief that a salt of magnesia was present.

From the result of these few and imperfect experiments, we may venture to enumerate the following as among the principal constituents of the Cornus Florida: 1. Gum; 2. Resin; 3. Tannin; 4. Gallic Acid; 5. Oil; 6. Fatty Matter; 7. a Crystalline Substance; 8. Bitter Extractive; 9. Wax; 10. Red Coloring Matter; 11. Lignin; 12. Potassa; 13. Iron. To which may be added Salts of Lime and Magnesia—*Cornus Florida, by James Cockburn, Jr. Extract from Thesis. Phil. Col. of Phar. American Journal of Pharmacy. July,* 1835. *New Series. Vol.* 1. *Pages* 111–114.

Dr. D C. O'Keefe, whilst a student of medicine in the Medical College of Georgia, published a valuable article on the chemical constitution and febrifuge properties of dog-wood bark, in which he states that, with the assistance of Dr. Robert Campbell, he had determined upon and conducted the following process for obtaining cornine:

Pulverize two pounds of the well dried bark of the root; separate its tannin with sulphuric ether, and filter. Macerate the separated bark in alcohol for two days to extract its resin and cornine. Pour off the alcohol, and precipitate the resin with water. Filter off the resin, and precipitate the cornine from the liquor with a solution of subacetate of lead. Separate the subacetate of lead from the solution by passing a current of sulphuretted-hydrogen gas through it. Filter and evaporate the fluid down to the cornine.

This substance is possessed of decided acid properties, having a well marked acid reaction. It is of a dark straw color, very bitter and astringent.—*Southern Medical and Surgical Journal. January*, 1849. *Pages* 6–7.

Dr. O'Keefe cites the testimony of Professor Gegier, of Heidleberg, as confirmatory of the results of his examination of the acid properties of cornine.

It is evident, from the discrepancies in the statements and views of these various observers, that the analyses of dog-wood, thus far published, are not sufficiently thorough and accurate, and that the profession needs more extended and definite information with reference to the chemical and physical properties of this valuable indigenous plant.

*Medical Properties and Uses.*—The bark of the dogwood has been known and successfully used in the treatment of intermittent fevers for more than one hundred years.

Upon the human body the bark of the Cornus Florida acts as a tonic, astringent and antiperiodic, and resembles in its general effects Peruvian bark.

Dr. Benjamin Smith Barton, in the *Philadelphia Medical and Physical Journal*, of 1805, says: "The bark of the Cornus Florida, or common Dogwood, does more than support its former reputation. It was used with much success in the generally prevailing intermittents of Maryland and Virginia in 1804. By some respectable practitioners, it was deemed but little inferior to good Peruvian bark." Page 181.

Dr. Walker, by numerous experiments with it upon the healthy system, determined that it uniformly increased the force and frequency of the pulse, and augmented the heat of the body. He instituted collateral experiments with the Peruvian bark, and found that both its internal and external effects agreed with those of the Cornus.

Dr. Gregg, of Bristol, Pennsylvania, states that after employing the Cornus Florida for nearly twenty-three years in the treatment of intermittents, he was satisfied that it was not inferior to Peruvian bark, and that he had found it uniformly beneficial as a tonic in cases of debility. Among the number of cures by this medicine was that of his own case. Dr. Gregg estimated thirty-five grains of it equal to thirty grains of Peruvian bark, and observed that the only inconvenience accompanying its use was that, if taken within a year after being stripped from the tree, it sometimes occasioned acute pains in the bowels; but this evil was remedied by adding to it five grains of Virginia snake root (aristolochia serpentaria.) He recommends the bark as being in the best state after it has been dried a year.

In an intermittent fever, which prevailed many years ago in West Jersey, it is said to have proved, generally speaking, more beneficial than Peruvian bark.

Drs. Jacob Bigelow, S. G. Morton, R. Coates, and many other medical men, have employed this bark with advantage in intermittents and in debilitated states of the system, accompanied with loss of appetite and indigestion. I have myself used it with good success in the treatment of our climate fevers.

In the Southern part of Georgia I have known the planters to employ it extensively amongst their people, in combination with the wild cherry bark and wild horehound, (eupatorium pilosumo) not only in the treatment of intermittent fever, but also in colds and dropsies, and in all cases of debility accompanied with loss of appetite and indigestion.

Dr. B. S. Barton states that a decoction of the dogwood bark was found very useful in a malignant disorder of horses called "yellow water."

Dr. D. C. O'Keefe, in the article previously referred to, gives an interesting account of the physiological, as well as the therapeutic action of the extract of dogwood, and supports his views by fifteen accurately detailed cases of intermittent fever.

In order to ascertain with precision the effects of large doses of the extract on the system in a physiological state, Dr. O'Keefe instituted the following experiment upon himself:

10 A. M., first dose, 30 grs. extract; pulse previous to taking it, 72.

11 A. M., second dose, 30 grs.; pulse intermittent, 72–76; temperature of surface somewhat augmented; general perspiration; a sense of fullness, and slight dull pain over the frontal eminences, much increased on flexing the head forward and downward; uneasy feelings in the stomach and bowels.

12 M., third dose, 30 grs.; pulse 76, not intermittent, but somewhat depressed; sensation in the head uniform. On taking this dose, a sense of warmth was felt in the stomach, and radiated over the surface of the trunk.

1 P. M., fourth dose, 30 grs.; pulse 76, and regular; pain in the head augmented, and extended down the forehead to the eye-lids, with a disposition to sleep; slight oppression in the precordia.

Eating dinner neither mitigated nor heightened the dull headache, which continued the same throughout the day. At night tendency to sleep much more urgent. Retired early; slept well during the night, and arose in the morning free from any uneasy sensations whatever.—*Southern Medical and Surgical Journal*, *January*, 1849. *Pages* 10 *and* 11.

The discrepancies between the effects observed by Dr. O'Keefe and Dr. Walker may have been due to the fact that the former used the extract and the latter the bark. Be this as it may, it is nevertheless true that the profession needs an extended series of experiments upon the action of the various preparations and constituents of the Cornus Florida. Until these data are supplied, it would be worse than useless to attempt any critical analysis and description of its physiological effects.

Dr. O'Keefe not only substantiates the testimony of various physicians to the great value of dogwood in the treatment of malarial fever, but he also establishes the fact that the extract has no tendency whatever to disturb the stomach and bowels. This is important, for the alleged tendency of the Cornus to disturb the stomach and bowels mentioned by so many writers has exerted no little influence in causing this valuable remedy to remain neglected.

According to Mr. Carpenter, the Cornus Florida yields a beautiful extract, resembling very closely that of Cinchona, differing, however, in its sensible characters from the extract of the superior species of Peruvian bark, by being less bitter and more astringent. The following is the most eligible mode for preparing this extract:

Evaporate in a sand or water bath a tincture of the bark, made by digesting it in proof spirits in the proportion of two ounces of the former to a pint of the latter, suffering it to stand for at least a week before straining; occasionally during this time submitting it for a few hours to a moderate heat, and thereby facilitating the solution. This extract, from its most prominent and sensible characters, is unquestionably much more active than the common extract of Carthagena bark, and is a preparation admirably adapted in all cases where the Cornus may be employed with advantage; and in consequence of being a concentrated preparation, separated from the ligneous and insoluble portions, and containing less gum and mucous matter, (which constitutes so large a portion) is certainly much preferable to the crude substance, and no doubt will be resorted to by many country practitioners as a useful expedient, particularly in those places where this article is in profusion, and where bark of a good quality is frequently very scarce, and sometimes even unknown.—*Essays on Materia Medica, &c., by G. W. Carpenter. Pages* 203–204.

The extract thus prepared has been exhibited with success by several practitioners, in the same doses as the alcoholic extract of Cinchona.

Dose of extract of Cornus Florida, from gr. x to ʒ ii, repeated as often as the case demands. Dose in powder, from twenty to thirty grains, to be repeated according to circumstances. It may also be given in decoction, made with an ounce of the bark to a pint of water, of which the dose is from an ounce to two ounces. In some parts of the country the ripe berries infused in brandy have been used as bitters, and the infusion of the flowers is said to form a good substitute for chamomile tea. A decoction of the buds and twigs has been thought to agree better with weak stomachs than the other preparations.

During the recent war, in both civil and military practice, I have used the decoction and tincture of dogwood to a considerable extent, and found the remedy of value in the treatment of malarial fever. In the severe cases the paroxysm was arrested with sulphate of quinine, and the patients were then put upon the dogwood, to secure the tonic as well as the antiperiodic properties. I also employed in military practice in the treatment of intermittent fever, with most satisfactory results, a mixture composed of the tincture of dogwood, nitric acid and common salt. The following is the formula most generally employed:

℞—Saturate tincture of dogwood bark, f. ℥ xij.; nitric acid (concentrated), f. ʒ i.; common salt (chloride of sodium), ℥ i. Mix tablespoonful in cup of water every four hours, sucked through a quill.

Under this preparation not only was the recurrence of the chills prevented, but the sallow, jaundiced complexion of the soldiers, who had long been exposed to the action of malaria, assumed the clear hue of health. Both the nitric acid and the common salt in this mixture were also efficient agents in breaking up the paroxysms, and in causing such an increased action of the liver and kidneys as removed the effete compounds resulting from the prolonged action of the malarial poison.

As far as my personal investigations extend, I was led to the belief that the tincture of dogwood possesses decided prophylactic powers against malarial fever.

The compound tincture of dogwood was issued by the Medical Purveyors to the Confederate troops serving in damp, swampy, marshy, malarious regions with good effects in protecting the troops against malaria.

Thus the Eutaw (25th South Carolina) regiment, whilst it was encamped upon James' Island, in a locality notorious for the prevalence of malarial fevers of the severest character during the summer and fall months. This regiment had a mean strength of near eight hundred officers and men. During the summer and autumn of 1862, as large a proportion as one-third of the mean strength were at times upon the sick list with the various forms of malarial fever.

The assistant surgeon of this regiment, J. W. Warren, of South Carolina, communicated to the author, during his inspection of the sick upon James' Island, some interesting facts upon the prophylactic powers of certain indigenous remedies.

A compound tincture, or medicated whisky, prepared by the Medical Purveyor from the dogwood, cherry, poplar and willow barks, was administered daily, in the proportion of one-half to one gill to each man during two weeks in the month of September, 1862. Under the use of this tonic mixture the number of new cases of malarial fever diminished one-half, although as the autumnal season advances upon James' Island malarial fevers increase in number and severity. The supply of this medicated whisky being limited, at the end of two weeks it was exhausted, and in the course of eight days the cases of malarial fever had increased from thirty-six to eighty. A fresh supply having been obtained, its use was again commenced, and in the course of five days the number of cases of malarial fever fell to the original number.

In the absence of quinine, a strong tincture of these barks was used with good effects in the treatment of malarial fever. I requested Assistant Surgeon Warren to continue these experiments, with certain variations, designed to determine the active and the inert ingredients in the tincture, and also indicated a plan by which the prophylactic powers of dogwood might be fairly tested.

Dogwood received the special attention of the Surgeon-General, S. P. Moore, of the Confederate States army, and of the Medical Purveyors, at an early period in the war, as will be seen from the following official papers:

CIRCULAR.

CONFEDERATE STATES OF AMERICA,
SURGEON-GENERAL'S OFFICE.
Richmond, Va., Dec. 5, 1862.

———, *Medical Purveyor C. S. A.:*

SIR—Below you will find a formula for a compound tincture of the indigenous barks, to be issued as a tonic and a febrifuge, and substituted, as far as practicable, for quinine.

Very respectfully, your obedient servant,
SAMUEL P. MOORE, S. G., C. S. A.

Dried dogwood bark 30 parts.
Dried poplar bark 30 parts.
Dried willow bark 40 parts.
Whisky 45 degrees strength.

Two pounds of the mixed barks to one gallon whisky. Macerate fourteen days and strain.

Dose one fluid ounce (f. ℥j) three times a day.

CIRCULAR NO. 12.

CONFEDERATE STATES OF AMERICA,

PURVEYOR'S OFFICE,

Richmond, Va., August 22, 1862.

———, *Medical Purveyor, C. S. A.:*

SIR—Although no orders have been issued to that effect, some of the Purveyors appear to be under the impression that they should make a mixture of the indigenous barks (dogwood, &c.,) and whisky. The arrangement intended by the Surgeon-General and Commissary-General is, that the Commissary Department shall furnish the whisky to the troops, giving each man one drink a day. The Purveying Department was to furnish the barks to mix with the whisky, to make a species of army bitters, as a preventive against malaria, &c. The arrangement is merely an issue of whisky by the Commissary Department to the troops, and the Purveying Department furnish the bark to mix with it. This office has not as yet been instructed whether the mixture is to be made at the Purveying depot or at the Commissary depot. Therefore, whisky will not be issued in any other than the medical preparations that have been, or may be, ordered as regular issues.

Very respectfully, your obedient servant,
E. W. JONES, Chief Med. Purv. C. S. A.

CIRCULAR NO. 19.

CONFEDERATE STATES OF AMERICA,
MEDICAL PURVEYOR'S OFFICE,
Richmond, Va., November 1, 1862.

———, *Medical Purveyor, C. S. A.:*

SIR—Your attention is called to the following extract from Carpenter's Essay on the Materia Medica:

* * * "The Cornus Florida yields a beautiful extract, resembling very closely that of cinchona, differing, however, in its sensible character from the extract of the superior species of Peruvian bark, by being less bitter and more astringent.

"The following is the most elegant mode for preparing this extract: Evaporate in a sand or water bath a tincture of the bark made by digesting it in proof spirits, in the proportion of two ounces of the former to a pint of the latter, suffering it to stand at least a week before straining, occasionally during the time submitting it for a few hours to a moderate heat, and thereby facilitating the solution.

"This extract, from its most prominent and sensible character, is unquestionably much more active than the common extract of Carthagena bark, and is a preparation admirably adapted in all cases where the Cornus may be employed with advantage, and in consequence of being a concentrated preparation, separated from the ligneous and insoluble portion, and containing less gum and mucous matter (which constitute so large a portion), is certainly much preferable to the crude substances, and no doubt will be resorted to by many country practitioners as a useful expedient, particularly in those places where this article is in profusion, and where bark of a good quality is frequently very scarce, and sometimes even unknown.

"The extract thus prepared has been exhibited with success by several practitioners, in the same doses as the alcoholic extract of cinchona.

"Dose of extract of Cornus Florida from ten grains to two drachms, repeated as often as the case demands."

This preparation appears to be a desirable one, and you will accordingly include it among those to be prepared for issue. Very respectfully, your ob't serv't,

E. W. Jones, Chief Med. Purveyor.

Office Medical Purveyor,

Montgomery, Ala., Feb. 11, 1863.

———, *Medical Purveyor, C. S. A.:*

Sir—Yours of the 9th inst., has been received. I can send you thirty-five pounds spts. nitre dulce. I am preparing it, but will be out of nitric acid in a day or two. If you can send me some nitric acid I can manufacture spts. nitre for you. I have a few pounds of aqua ammonia to spare, and can make you some if you can procure for me muriate of ammonia. I can send you fluid extract sarsaparilla, tinct. muriate iron., fluid extract of blackberry, tinct. of dogwood, poplar and willow, solid extract podophyllum, syrup of squills, solid extract of dogwood, syrup wild cherry. All these I am manufacturing in considerable quantity. I am now making aether sulph. in order to make tannin. and chloride of lime for making chloroform, both of which I shall manufacture this month.

The fluid extract of blackberry I can recommend as a capital astringent in dysentery and diarrhœa. The extract of podophyllum is a safe and thorough purge. The extract of dogwood is a good substitute for quinine. The tinct. of dogwood, poplar and willow is used in the hospitals here in large quantities. All these preparations are made with care. Your obedient servant,

WM. H. ANDERSON, Med. Purveyor.

The following observations upon the medicinal properties of the Cornus Florida (dogwood) appeared in a valuable work, prepared by Surgeon Francis Peyre Porcher, M. D., of Charleston, South Carolina, and published by order of the Surgeon-General, C. S. A., 1863:

*Cornus Florida* (*Dogwood*).—This well known plant possesses tonic and antiperiodic properties very nearly allied to those of cinchona. In periodic fevers, one of the most valuable of our indigenous plants. Dr. Gregg states that after employing it for twenty-three years in the treatment of intermittent fevers, he was satisfied that it was not inferior to Peruvian bark. Generally given in conjunction with laudanum. It also possesses antiseptic powers. In the recent state it is less stimulating than the cinchona bark, but it affects the bowels more. The dried bark is the preferable form. The fresh bark will sometimes act as a cathartic. It is more stimulating than thoroughwort (eupatorium), and, therefore, is less applicable during the hot stages of the fever. * * In our present need of astringent antiperiodics and tonics, the dogwood bark powdered will be found the best substitute for the Peruvian. Internally and externally, it can be applied wherever the cinchona barks were found servicable. The dogwood bark and root in decoction, or in form of cold infusion, is believed by many to be the most efficient substitute for quinine, also in treating malarial fevers. Certainly it might be used in the cases occuring in camp to prevent the waste of quinine, as it can be easily and abundantly procured.

Dr. Richard Moore, of Sumpter district, informs me that he not only finds it efficient in fevers, but particularly useful, with whisky or alcohol, in low forms of fevers and dysentery occurring near our river swamps.

During convalescence, when an astringent tonic is required, this plant supplies our need. See Eupatorium (Boneset) and Liriodendron. These, with the blackberry and chinquapin as astringents, the gentians and pipsissiwa as tonics and tonic diuretics, the sweet gum, sassafras and beni for their mucilaginous and aromatic properties, and the wild jalap (podophylum) as a cathartic, supply the surgeon in camp with easily procurable medicinal plants, which are sufficient for almost every purpose. Nitrate and bi-carbonate of potash are most required, and, with calomel, may be procured from abroad. Our supply of opium can be easily procured by planting the poppy and incising the capsules. Every planter could raise a full supply of opium, mustard and flaxseed. The wood of the dogwood, like the willow, is preferred in making gunpowder. See Salix.

A tonic compound, as advised by the herbalists, is made with the bark of the dogwood, columbo (frasera), poplar, each six ounces; bark of wild cherry, six ounces; leaves of thoroughwort, four ounces; cayenne pepper, four ounces, sifted and mixed. Dose, a teaspoonful, in warm or cold water, repeated.

It is stated in the Newbern *Progress* that a ripe dogwood berry taken three times a day, before meals, will cure ague and fever.

My friend, Professor F. A. P., contributes the following to the Charleston *Courier:*

[The dogwood bark, powdered, may be used in place of the Peruvian mentioned.]

*Dutch Remedy for Fever and Ague.*—As quinine is very scarce, it may not be unprofitable, both to our armies and private families, to revive the memory of an ancient remedy, which was in almost universal use before the introduction of the former drug. It was known by the name which heads this article, and has been used from time immemorial among the Huguenot families of the Santee, among whom there is a tradition that it was brought to this country by the ancestor of one of the families, who was a physician. The remedy quoted below is copied from an old receipt book. Though not a professional man, I can speak for its efficacy when it was in vogue:

*The Recipe.*—Two ounces of Peruvian bark, two ounces of cream of tartar, sixty cloves.

*Manner of Using It.*—These ingredients are to be rubbed together in a mortar. The mixture to be divided into twenty-four doses, four of which (mixed in water) are to be given the first day, four on the second, and two on every succeeding day, until the whole shall have been taken. It is probable that the disease will be arrested on the second or third day; but the object in taking the whole prescription is to complete the cure by its tonic property.

The berries of the dogwood have also been highly recommended; given as a remedy for fever in place of quinine (1862). One or two given in form of pill.—*Resources of the Southern Fields and Forests, Medical, Economical and Agricultural, &c., by Francis Peyre Porcher, M. D., Surgeon, P. A. C. S.* 1863. *Pages* 59–62.

NO. 3.—CORNUS CIRCINATA, *wild* (ROUND-LEAVED DOGWOOD), AND CORNUS SERICEA, *wild* (SWAMP DOGWOOD).

The ten species of Cornus indigenous to the United States are all supposed to possess similar medicinal properties. With the exception of the Cornus Florida, the two now under consideration have been most carefully investigated. Our knowledge, however, of both their chemical and medicinal properties is not only more imperfect than that of the Cornus Florida, but is vague and meagre. Professors Mason and Ives appear to have been the first to introduce the Cornus Circinata into medical practice. They recommend it very highly for its astringent and tonic properties, and affirm that they have successfully used it in intermittent fevers and dysentery. Mr. Carpenter announced that the alkaloid principle, cornine, exists in this species of Cornus.

The alcoholic extract appears to be the most eligible mode of using this article. The extract is prepared in the same manner with that of the Cornus Florida. It possesses more astringency, and is, therefore, better adapted to the treatment of dysentery. As this plant appears to be rare in most of the Southern States, it is not likely that it will ever be extensively employed, especially as the Cornus Florida is not only more abundant, but also fully as efficient. The bark of the Cornus Sericea (swamp dogwood) was found by Dr. Walker to be equal to that of the Cornus Florida, and but little inferior to the common pale Peruvian bark in the treatment of intermittents. It forms a beautiful tincture with proof spirits.

As the swamp dogwood inhabits the North American continent from Canada to Florida, growing in moist woods, in swamps, and on the border of streams, especially in the mountains, it is well worth the attention of the physicians of the United States.

The dose and modes of preparation and administration are the same with those of the Cornus Florida.

